# Spontaneous Coronary Artery Dissection in Women

**DOI:** 10.31083/RCM44459

**Published:** 2025-12-16

**Authors:** Fabiana Lucà, Iris Parrini, Alaide Chieffo

**Affiliations:** ^1^Department of Cardiology, Grande Ospedale Metropolitano, 89128 Reggio Calabria, Italy; ^2^Cardiology Unit, Koelliker Hospital, 10134 Torino, Italy; ^3^Interventional Cardiology Unit IRCCS San Raffaele Scientific Institute, 20132 Milan, Italy

Spontaneous coronary artery dissection (SCAD) has been recognized as an acute coronary condition of non-atherosclerotic and non-traumatic origin that may result in an acute myocardial infarction (AMI) with non-obstructive coronary arteries (MINOCA), arrhythmic events, and sudden cardiac death (SCD) [1, 2, 3, 4, 5, 6, 7, 8, 9, 10].

It has been reported that SCAD has a higher prevalence in females, particularly in those aged 44–53 years [1, 2]. However, it may occur in both sexes, although it is less likely to be observed in males [3, 4, 5, 6, 7, 8, 9, 10, 11]. Nonetheless, there seems to be a general consensus of opinion that SCAD is a cause of acute coronary syndrome (ACS) from adolescence to advanced age [12], accounting for approximately one-quarter of an AMI in young females [13, 14].

Another point worth noting is pregnancy-associated SCAD (P-SCAD). It is widely understood that 15–20% of ACS in pregnancy can be due to SCAD [15, 16]. This may be related hormonal therapy, as well as physical and emotional stress, which may increase the risk of initial or recurrent events [17].

It has been found that patients experiencing SCAD are less likely to present with traditional cardiovascular (CV) risk factors in comparison with those with ACS due to atherosclerosis [18]. It should also be mentioned that any coronary artery may be affected, although the left anterior descending artery and mid-segment vessels are most commonly involved [19].

In terms of underlying pathophysiological mechanisms, the concept that the development of an intramural hematoma may lead to separation of the arterial wall layers and subsequent intimal dissection has been supported [20]. This may cause complete vessel occlusion or decompress into the lumen, resulting in the restoration of distal flow (**Supplementary Fig. 1**). In contrast, rupture of the vasa vasorum may be implicated. Several predisposing factors have been proposed, including sex, hormonal fluctuations, pre-existing arteriopathies, and genetic syndromes such as Marfan syndrome and Loeys-Dietz syndrome, which weaken the arterial wall [19, 21].

Hormonal fluctuations, such as the marked increase in estrogen and progesterone during pregnancy, the abrupt changes in the peripartum period, and the decline in estrogen after menopause, may influence vascular integrity through multiple mechanisms [18]. Elevated progesterone levels during pregnancy can alter collagen composition and reduce the density of medial smooth muscle cells, thereby weakening the arterial wall. Estrogen fluctuations can modulate endothelial function, nitric oxide bioavailability, and matrix metalloproteinase activity, leading to changes in extracellular matrix turnover. These molecular alterations, in combination with increased hemodynamic stress and inflammatory mediators, may predispose to medial degeneration and facilitate the formation of intramural hematomas. These mechanisms provide a plausible explanation for the higher prevalence of SCAD in females and the observed associations with pregnancy and the early postpartum period [18].

It has been reported that environmental influences, inflammatory diseases, sympathomimetic drugs (e.g., cocaine, amphetamines), and immunosuppressants such as cyclosporine, tacrolimus, and high-dose corticosteroids may be related to SCAD’s occurrence [16].

Furthermore, a strong association between SCAD and fibromuscular dysplasia (FMD) has been reported. The involved arteries angiographically appear as a “string-of-beads”, due to alternating fibromuscular ridges, which leads to stenosis and aneurysm formation [19, 22]. P-SCAD has been attributed to gestational hormonal fluctuations, particularly elevated circulating progesterone levels that may compromise vessel integrity. This suggests that increased hemodynamic stress and delivery can precipitate arterial dissection. It has been found that there is an increased risk of recurrent SCAD, and patients should be counselled about the potential danger SCAD in future pregnancies [23].

Although changes in hormones is an important predisposing factor, given SCAD’s predominance females, a significant difference in the incidence between nulliparous and multiparous women has not been definitively reported [24, 25, 26]. In addition, a significant number of cases (55%) occur post-menopause. This is juxtaposed with the hypothesis that other mechanisms may be involved in SCAD [17].

It has been recommended that females with a history of P-SCAD undergo a comprehensive CV assessment before attempting a new pregnancy [27, 28, 29]. Pre-pregnancy evaluation should include detailed coronary imaging, preferably Coronary Computed Tomography Angiography (CCTA) or Invasive Coronary Angiography (ICA), in stable patients, ideally performed at least 6–12 months after the index event to document complete arterial healing [30]. Additional vascular imaging should be considered to exclude extracoronary arteriopathies, particularly fibromuscular dysplasia [31]. The suitability for pregnancy should be based on the absence of residual dissection, significant stenosis, ventricular dysfunction, and achieving optimal control of CV risk factors.

During pregnancy, a multidisciplinary team, including cardiology, maternal-fetal medicine, and anaesthesiology, should coordinate care. Strategies to mitigate recurrence risk include limiting excessive physical exertion, avoiding severe hypertension, and planning delivery in a tertiary center with immediate access to cardiology and cardiac surgery services [32]. Vaginal delivery with careful hemodynamic monitoring is generally preferred unless obstetric or cardiac indications require caesarean section. Close postpartum surveillance is critical, as the risk of recurrence is highest in the first weeks after delivery [33].

SCAD may present as an ST Elevation Myocardial Infarction (STEMI), with ventricular arrhythmias and cardiogenic shock occurring in approximately 20–50%, 3–5%, and 2% of cases, respectively. SCAD may also manifest as an Non-ST Elevation Myocardial Infarction (NSTEMI) [34, 35]. Prompt recognition and accurate diagnosis of SCAD is SCAD.

However, despite the rise in recognition of SCAD as a significant cause of ACS in females, both clinical awareness and management, in the short and long-term, remain suboptimal.

SCAD can be angiographically classified into four types [21] (**Supplementary Fig. 2**).

The angiographic classification of SCAD has important clinical implications [36]. Type 1 SCAD, characterized by the presence of an intimal flap or double lumen, is often associated with more extensive dissections and, when accompanied by persistent ischemia or impaired coronary flow, may require urgent revascularization. Type 2 SCAD, the most common form, typically manifests as a long, diffuse stenosis and has a higher probability of spontaneous healing, thus favoring conservative management in stable patients. Type 3 SCAD may resemble focal atherosclerotic disease, which increases the risk of misdiagnosis; its short segment involvement can facilitate Percutaneous Coronary Intervention (PCI) when clinically necessary, although intracoronary imaging is often required for confirmation. Type 4 SCAD presents as a total occlusion and frequently mimics thrombotic occlusion in an STEMI, and is associated with lower baseline Thrombolysis In Myocardial Infarction Risk Score (TIMI) flow and potentially greater myocardial injury. While robust comparative prognostic data are still limited, vessel location, dissection length, and baseline coronary flow, features which are variable among the various types of SCAD, may influence patient outcomes, the risk of recurrence, and the procedural success of revascularization. Integrating the angiographic features into the overall clinical assessment may therefore improve risk stratification and guide personalized management strategies [37].

It is important to mention that SCAD may be missed on invasive coronary angiography (ICA); thus, intravascular ultrasound (IVUS) and optical coherence tomography (OCT) are essential for diagnosis. Double lumen or “flap” images on angiography suggest false lumen opacification. Coronary Computed Tomography Angiography (CCTA) can be an effective non-invasive alternative for monitoring arterial healing during follow-up. When the coronary ostia are involved, it is essential to rule out aortic dissection [38]. The choice of imaging modality in SCAD depends on the clinical scenario and the specific diagnostic objectives. In the acute phase, IVUS and OCT offer high spatial resolution for detailed assessment of the arterial wall and identification of intramural hematomas or intimal tears. OCT provides superior axial resolution but requires contrast injection, which may exacerbate dissection in unstable patients [39]. IVUS, while offering lower resolution, has the advantage of avoiding the need for high-pressure contrast delivery and is preferable in hemodynamically compromised individuals. During follow-up evaluation to study vessel healing, CCTA represents a non-invasive alternative, allowing serial assessments without the procedural risks of invasive imaging. However, its diagnostic accuracy may be reduced in cases of severe coronary calcification, small-caliber vessels, or complex coronary anatomy, where spatial resolution remains a limiting factor. In such instances, invasive imaging with IVUS or OCT may be required to clarify residual or recurrent abnormalities [40] (**Supplementary Fig. 3**).

According to the latest European Guidelines [41], the management of SCAD should be conservative. In most cases, spontaneous healing occurs within 30 days [42]. While conservative therapy remains the first-line approach in most cases, revascularization with Percutaneous Coronary Intervention (PCI) or coronary artery bypass grafting (CABG) should be considered in selected high-risk presentations [27]. These include patients with sustained hemodynamic instability, such as persistent hypotension (systolic blood pressure <90 mmHg), signs of systemic hypoperfusion, or evolving cardiogenic shock, as well as those with ongoing myocardial ischemia, evidenced by recurrent or refractory chest pain, dynamic ST-segment deviations on serial ECGs, or persistent elevation of high-sensitivity cardiac troponins despite optimal medical therapy. Additional triggers for intervention include sustained ventricular arrhythmias not controlled by pharmacological therapy and dissections involving the left main stem or proximal major coronary vessels with reduced antegrade flow and a large myocardial territory at risk. In such scenarios, the choice between PCI and CABG should be guided by lesion location, procedural feasibility, and the anticipated likelihood of restoring stable coronary perfusion without exacerbating the dissection. The goal of intervention is to restore coronary perfusion [43]. However, PCI can be technically challenging, with risks including hematoma propagation, difficulty in placing the guidewire within the true lumen, and stent mal-apposition following hematoma resorption.

This approach has been supported by data from the DISCO Registry on 369 patients with SCAD, which did not show significant differences in 2-year rates of major CV events between PCI and those receiving conservative therapy in terms of major CV events. However, there was a higher trend toward invasive treatment in the presence of more severe features—such as ST-segment elevation, proximal vessel involvement, and reduced coronary flow [44, 45]. In light of these findings, the potential safety and efficacy of a tailored, presentation-guided therapeutic strategy in SCAD has been suggested.

Regardless of whether cardiogenic shock is present, the use of mechanical circulatory support devices may be considered. Favorable clinical outcomes have been reported with the use of microaxial flow pumps [46].

Antiplatelet monotherapy, typically aspirin, has been reported to be correlated with more favorable outcomes The utility of statins is debated and should be reserved for patients with concurrent dyslipidemia. Beta-blockers and Angiotensin-Converting Enzyme (ACE) inhibitors are recommended, according to guidelines [41].

SCAD recurrence is not uncommon. SCAD recurrence, reported in approximately 20–30% of patients over a 5-year follow-up in the Canadian registry [47], appears to be influenced by a combination of anatomical, systemic, and lifestyle-related factors. Observational data suggest that multivessel coronary involvement at the index event, distal vessel location, and long-segment dissections may be associated with a higher risk of recurrence. Coexisting fibromuscular dysplasia is another important predictor, most likely reflecting a systemic arteriopathy that predisposes to repeated events. Hormonal influences, including exogenous estrogen or progesterone therapy, may contribute to a higher risk in susceptible females, although definitive causal relationships remain under investigation. Modifiable triggers—such as uncontrolled hypertension, exposure to extreme physical exertion, or significant emotional stress—should be addressed as part of the long-term management. Preventive strategies may include sustained beta-blocker therapy to reduce arterial shear stress, meticulous blood pressure control, avoidance of hormonal treatments when possible, and tailored exercise prescriptions within structured cardiac rehabilitation programs. While no intervention has been proven to eliminate the risk of recurrence, a multifaceted approach addressing both systemic and local predisposing factors is advisable.

Cardiac rehabilitation is strongly advised, preferably using tailored protocols that avoid isometric and high-intensity aerobic activity. Preventative strategies include avoiding emotional stressors, Valsalva maneuvers, hormonal therapy (including estrogen, progesterone, and β-HCG), and future pregnancies. Blood pressure control, and long-term beta-blocker therapy are indicated Magnetic Resonance Angiography is indicated to rule out FMD.

Coronary imaging should include assessment of the entire arterial tree, particularly in females with a desire for future pregnancy, given the risk of intracranial aneurysms.

A 5–7 day period of hospital observation is advisable, as this corresponds to the period during which complications are most likely to occur.

The long-term survival is promising, with only a 0.8% mortality reported at three years, based on evidence from the Canadian registry [47].

Due to the risk of recurrence, an adequate long-term follow-up, based on a holistic approach involving personalized pharmacological management and appropriate imaging surveillance, is highly recommended.

## Supplementary Material

Supplementary material associated with this article can be found, in the online version, at https://doi.org/10.31083/RCM44459.
