# Supplementary material for: Spontaneous Coronary Artery Dissection in Women
Source: Rev Cardiovasc Med. 2025 Dec 16;26(12):44459. doi: 10.31083/RCM44459 (PMC12780999; doi:10.31083/RCM44459)
Supplement: Supplementary file 1 [file 2153-8174-26-12-44459-s1.docx]

Supplementary Fig. 1. Pathophysiology of Coronary Artery Dissection (SCAD). SCAD development begins with the spontaneous formation of a hematoma within the tunica media of an epicardial coronary artery. This intramural hematoma results in the separation of the intimal and medial layers from the underlying arterial structure, creating a false lumen. The progression may result in either complete arterial occlusion—leading to myocardial ischemia—or spontaneous decompression through an intimal tear, which may allow partial restoration of coronary blood flow.


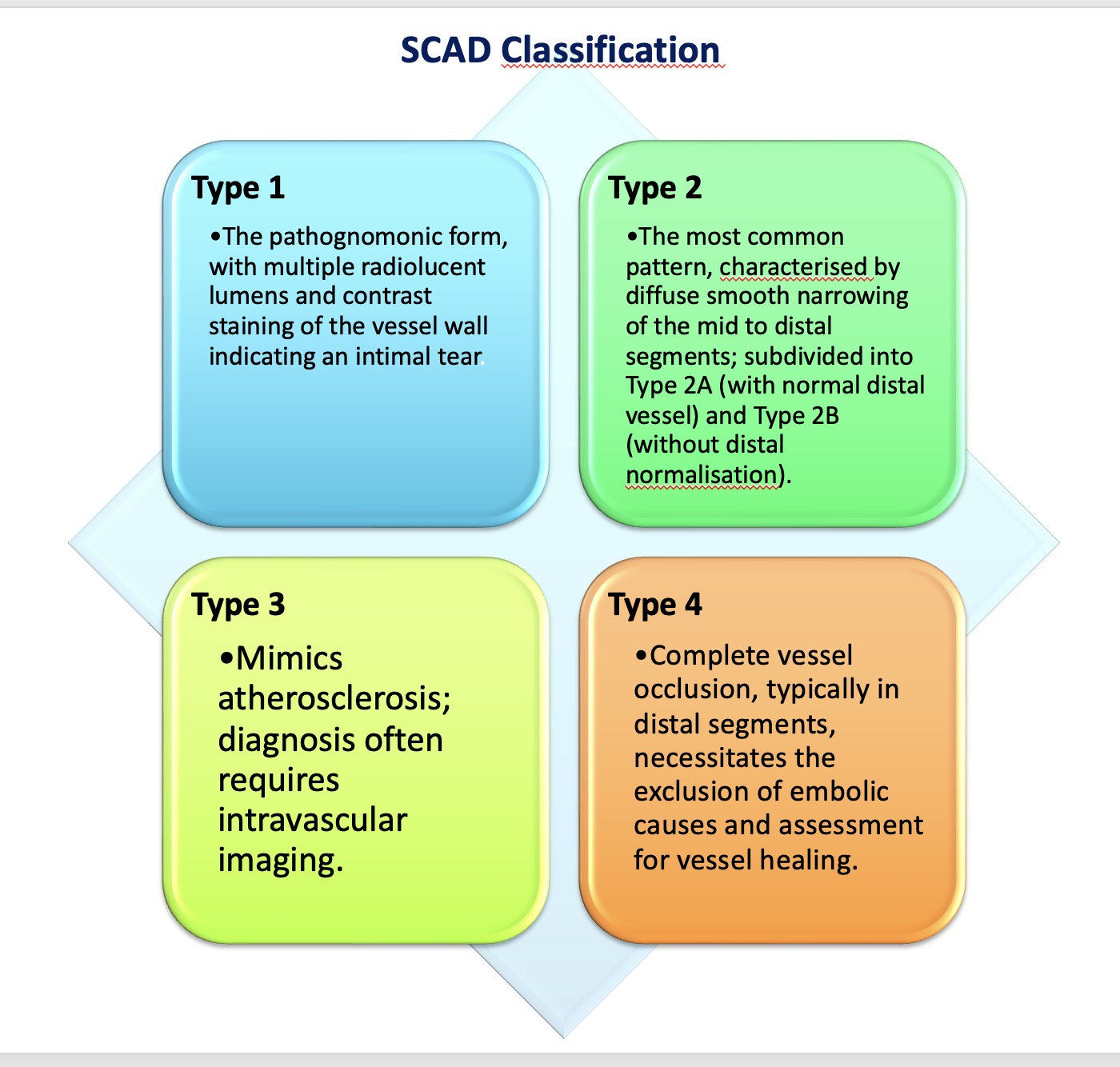


Supplementary Fig. 2. SCAD Classification.


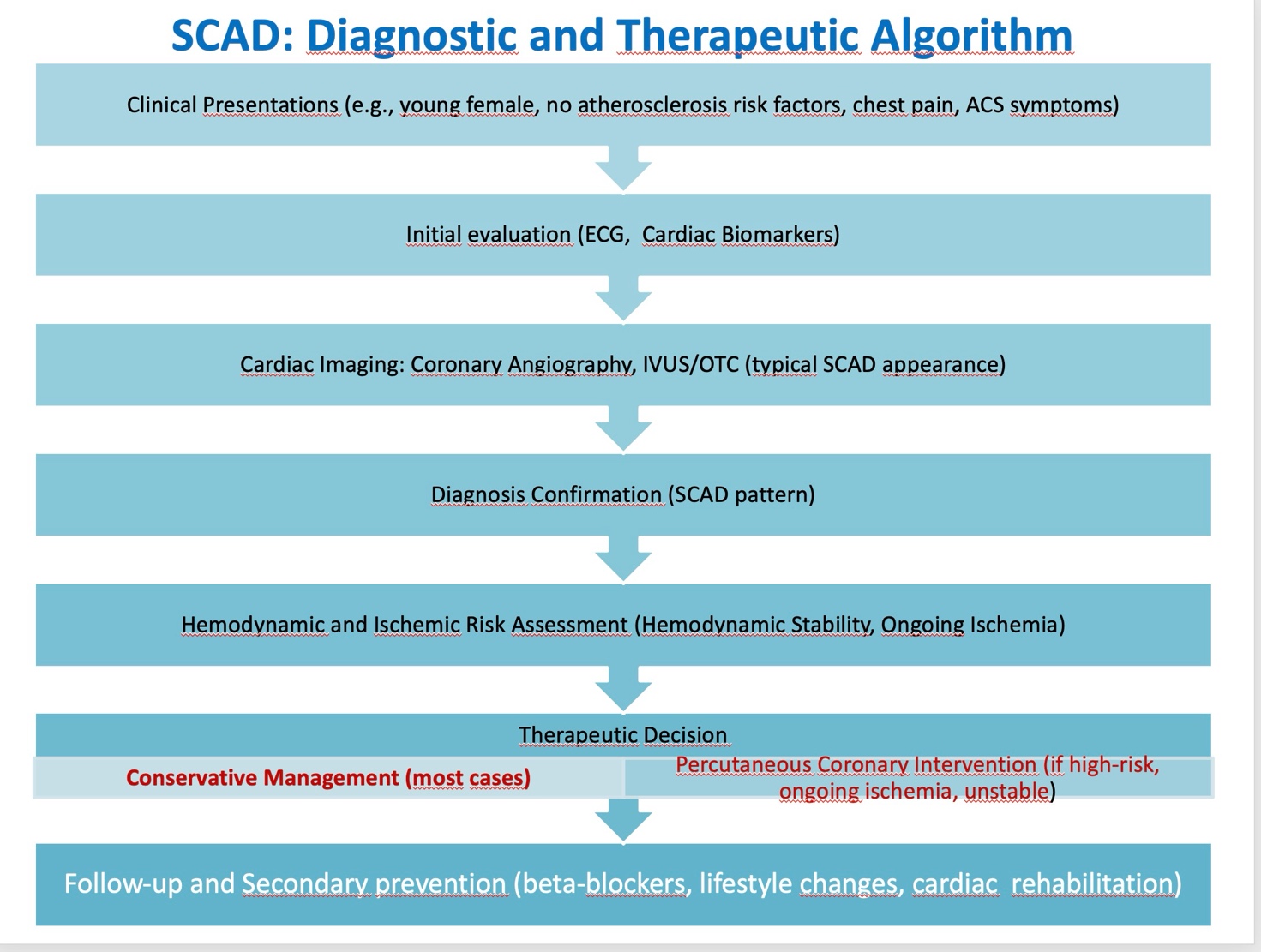


Supplementary Fig. 3. SCAD Diagnostic and Therapeutic Algorithm.
